# Micro RNA-98 suppresses interleukin-10 in peripheral B cells in patient post-cardio transplantation

**DOI:** 10.18632/oncotarget.16000

**Published:** 2017-03-08

**Authors:** Jiangping Song, Wenjun Su, Xiao Chen, Qian Zhao, Ningning Zhang, Mao-Gang Li, Ping-Chang Yang, Liqing Wang

**Affiliations:** ^1^ State Key Laboratory of Cardiovascular Disease, Fuwai Hospital, National Center for Cardiovascular Diseases, Chinese Academy of Medical Sciences and Peking Union Medical College, Xicheng District, Beijing, 100037, China; ^2^ The Research Center of Allergy and Immunology, Shenzhen University School of Medicine, Shenzhen, 518060, China

**Keywords:** B lymphocyte, interleukin-10, micro RNA, heart transplantation, cortisol

## Abstract

The immune tolerance to the transplant heart survival is critical. Regulatory B cells are one of the major immune regulatory cell populations in the immune tolerance. Micro RNAs (miR) can regulate the activities of immune cells, such as the expression of interleukin (IL)-10 by B cells. This study tests a hypothesis that micro RNA (miR)-98 plays a role in the regulation of interleukin (IL)-10 expression in B cells (B10 cell) after heart transplantation. In this study, the peripheral blood samples were collected from patients before and after heart transplantation. The expression of miR-98 and IL-10 in B cells was assessed by real time RT-PCR. An allograft heart transplantation mouse model was developed. We observed that after heart transplantation, the frequency of peripheral B10 cell and the IL-10 mRNA levels in peripheral B cells were significantly decreased, the levels of miR-98 were increased in peripheral B cells and the serum levels of cortisol were increased in the patients. Treating naive B cells with cortisol in the culture suppressed the expression of IL-10 in B cells, which was abolished by knocking down the miR-98 gene. Administration with anti-miR-98, or cortisol inhibitor, or adoptive transfer with B10 cells, significantly enhanced the survival rate and time of mice received allograft heart transplantation. In conclusion, the enhancement of serum cortisol affects the immune tolerant feature of B cells, which can be attenuated by anti-miR-98-carrying liposomes.

## INTRODUCTION

Heart transplantation is an optimal approach to rescue patients with advanced heart failure [1]. Since the donor hearts are alloantigens to the recipients, a specific immune response against the allograft hearts may be induced to cause the transplantation failure. Thus, to establish immune tolerance to the allograft hearts is necessary and critical for the allograft heart survival [2]. Upon the heart transplantation, immune suppressors are regularly administered to inhibit the possibility of allograft heart rejection. Because of the non-specific nature, administration with immune suppressors has some or severe side effects to the recipients [3]. Therefore, to efficiently establish immune tolerance to allograft hearts is of significance.

The immune tolerant system mainly includes immune tolerant cells and molecules. Regulatory T cells (Treg) and regulatory B cells (Breg) are the major immune tolerant cell fractions [4, 5]. Transforming growth factor (TGF)-β and interleukin (IL)-10 are the two important immune tolerant molecules [6, 7]. Although the generation of TGF-β and IL-10 in immune regulatory cells has been extensively studied [8, 9], the regulation of IL-10-producing B cells (B10 cells) in heart transplantation remains to be further investigated.

It is reported that psychological stress increases the levels of noradrenaline, adrenaline and cortisol in the peripheral blood system [10]. Cortisol is the endogenous glucocorticoid in human (corticosterone in rodents) that is released in response to various stressors such as physical injury and psychological stress [11]. Heart transplantation is a kind of stress on the patients. Whether such kind of stress affects the immune tolerant status has not been investigated.

It is reported that some micro-RNAs (miR) can break down immune tolerance [12]. MiRs are short (18–22 nucleotides in length) non-coding RNA chain. They function to silence RNA and regulate gene expression post-transcriptionally [13]. Recent reports indicate that miR-98 can suppress the IL-10 expression in B cells; such as Exposure B10 cells to IL-13 in the culture or over expression of miR-98 reduced the expression of IL-10 in B cells [14]; miR-98 inhibits IL-10 production and endotoxin tolerance in macrophages [15]. Whether miR-98 inhibits B10 cells in patients after heart transplantation has not been investigated. Under certain circumstance, B cells are capable of regulating the immune response to allograft transplantation [16]. Based on the above information, we hypothesize that the surgery-induced psychological stress increases cortisol release, which increases miR-98 expression in peripheral B cells to inhibit the development of B10 cells of patients after heart transplantation. In this study, we observed that less B10 cells and high levels of cortisol in the peripheral blood of patients after heart transplantation. Exposure to cortisol in the culture increased the expression of miR-98 in B cells, which inhibited the expression of IL-10 in B cells. Administration with anti-miR-98 liposomes significantly extended the allograft heart survival in mice.

## RESULTS

### Peripheral B10 cells are decreased in patients after heart transplantation

**Figure 1:**
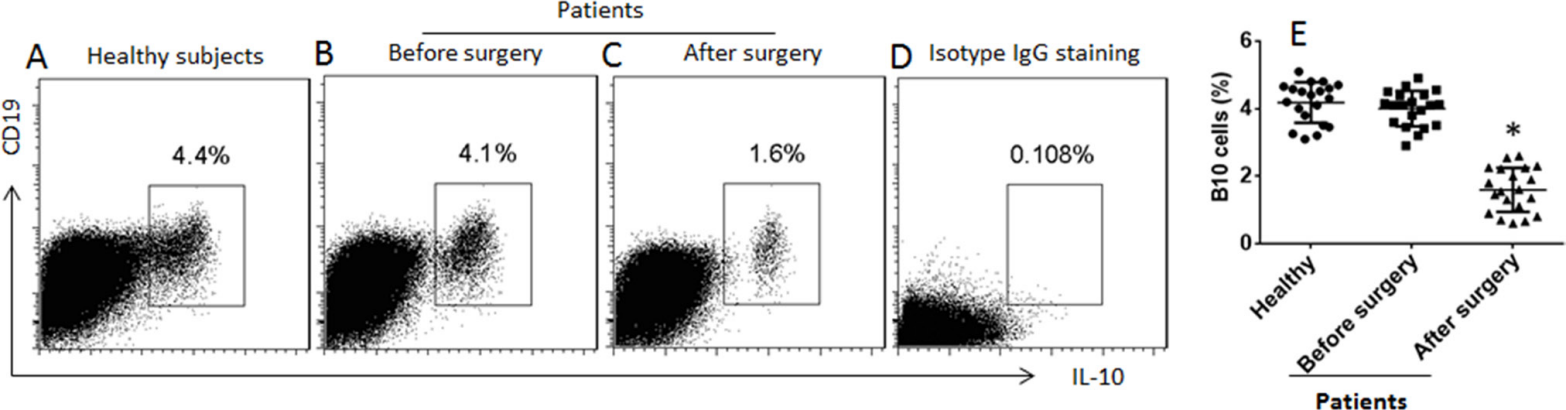
Assessment of peripheral B10 cells (**A**–**C**) the gated dot plots show the frequency of B10 cells in peripheral blood mononuclear cells of healthy subjects and patients one week before and one week after heart transplantation. (**D**) the isotype IgG staining results; used as a negative control. (**E**) the scatter dot plots show the data of A–C. The data were from 20 healthy subjects and 20 patients. Samples from individual patients were processed and analyzed separately. Data of bars are presented as mean ± SD. *p < 0.01, compared with the healthy subjects.

We collected the peripheral blood samples from patients a week before and a week after heart transplantation. Peripheral blood mononuclear cells (PBMC) were prepared and analyzed by flow cytometry. The results showed that the frequency of B10 cells in the patients was not different from the healthy control subjects, but significantly less after heart transplantation (Figure 1).

### Urinary cortisol levels are negatively correlated with IL-10 levels in peripheral B cells

**Figure 2:**
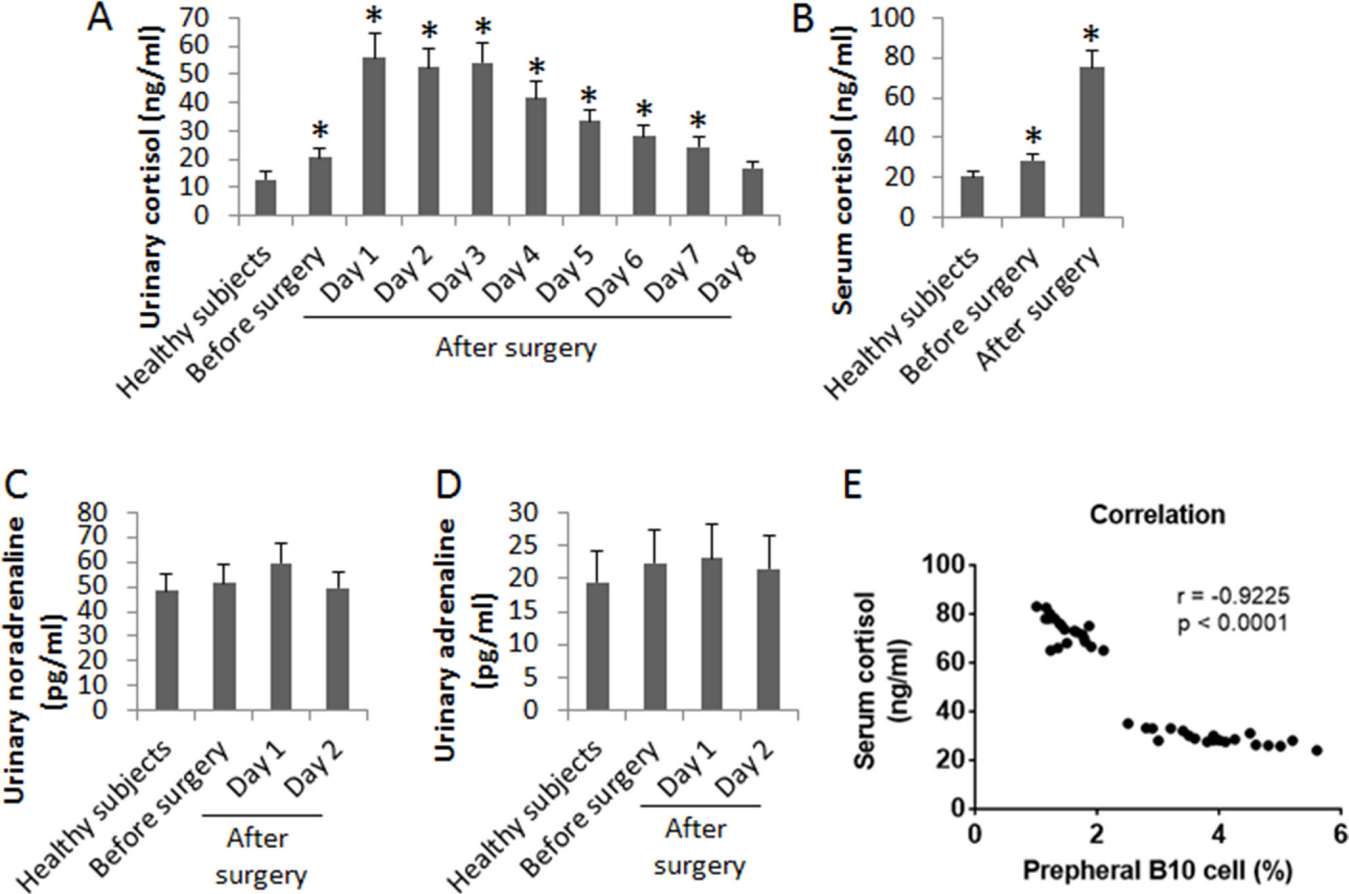
Assessment of “stress hormones” in patients after heart transplantation The bars indicate the urinary levels of cortisol (**A**), serum cortisol levels (**B**), urinary noradrenaline (**C**) and urinary adrenaline (**D**) in 20 healthy subjects, 20 patients before and after heart transplantation. Samples from individual patients were processed and analyzed separately. Data of bars are presented as mean ± SD. *p < 0.01, compared with the healthy subjects. E, the correlation between peripheral B10 cells and urinary cortisol levels (samples were collected one week before surgery and one week after surgery).

Since heart transplantation surgery is a kind of psychological stress on the body, we also assessed the levels of cortisol, noradrenaline and adrenaline in the urine of the patients. No significant difference was found in the levels of noradrenaline and adrenaline between patients and healthy subjects before surgery. However, the cortisol levels in the urine were higher in patients than that in healthy persons, which was further increased on the next day of surgery. It maintained at high levels for 3 days and then gradually declined and reached the control levels on day 8 after surgery (Figure 2A). We also assessed the serum levels of cortisol. The results showed that the levels of serum cortisol were also higher in patients than that in healthy persons, which was further increased in patients after surgery (on the 3rd day. Figure 2B). The urinary levels of noradrenaline (Figure 2C) and adrenaline (Figure 2D) in patients were slightly enhanced after surgery, but not reached the significant criterion. A correlation assay was performed with the data of the frequency of peripheral B10 cells and the serum cortisol. The results showed a negative correlation (*r* = −0.9225, *p* < 0.0001) between B10 cell frequency and the urinary cortisol levels. The results suggest that the enhancement of serum cortisol might be associated with the reduction of the frequency of peripheral B10 cells.

### Expression of miR-98 in peripheral B cells is positively correlated with urinary cortisol

**Figure 3:**
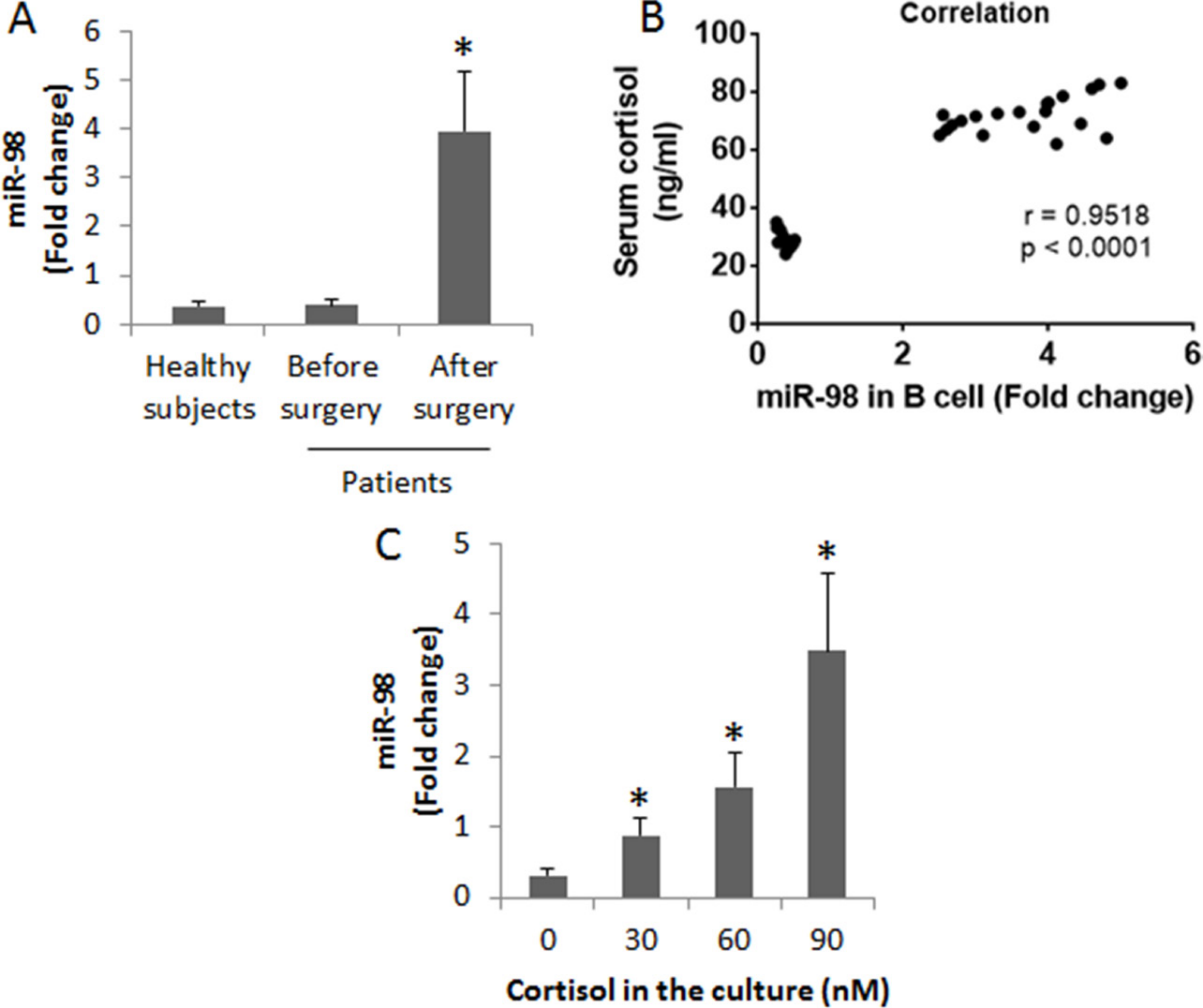
Assessment of miR-98 in B cells (**A**) the peripheral B cells were isolated from the blood samples collected from 20 healthy subjects and 20 patients a week before surgery and 3 days after surgery. The bars indicate the miR-98 levels in peripheral B cells of healthy subjects and patients. (**B**) the positive correlation between serum cortisol levels and the miR-98 levels in peripheral B cells. (**C**) miR-98 levels in B cells after exposure to cortisol in the culture for 48 h (the data B were summarized from 3 independent experiments). Data of bars are presented as mean ± SD. *p < 0.01, compared with the healthy subjects (A) or the dose 0 group (C).

Since miR-98 can bind the 5′-UTR area of the *il10* gene to repress the expression of IL-10 [15], we inferred that miR-98 might be increased in peripheral B cells and associated with the decrease in IL-10 expression. To test this, we analyzed the levels of miR-98 in peripheral B cells of patients. The results showed that the levels of miR-98 were increased in B cells after heart transplantation (Figure 3A). We then performed a correlation assay with the data of miR-98 in peripheral B cells and the serum cortisol levels. The results showed a positive correlation (*r* = 0.9598, *p* < 0.0001) between the expression of miR-98 in peripheral B cells and the cortisol levels (Figure 3B). The results implicate that cortisol might up regulate the expression of miR-98 in B cells. To test this, we treated B cells (from healthy subjects) with cortisol at gradient concentrations in the culture for 48 h. The B cells were analyzed by RT-qPCR. The results showed that cortisol did increase the expression of miR-98 in B cells in a cortisol concentration-dependent manner (Figure 3C).

### MiR-98 mediates cortisol-suppressed IL-10 expression in B cells

**Figure 4:**
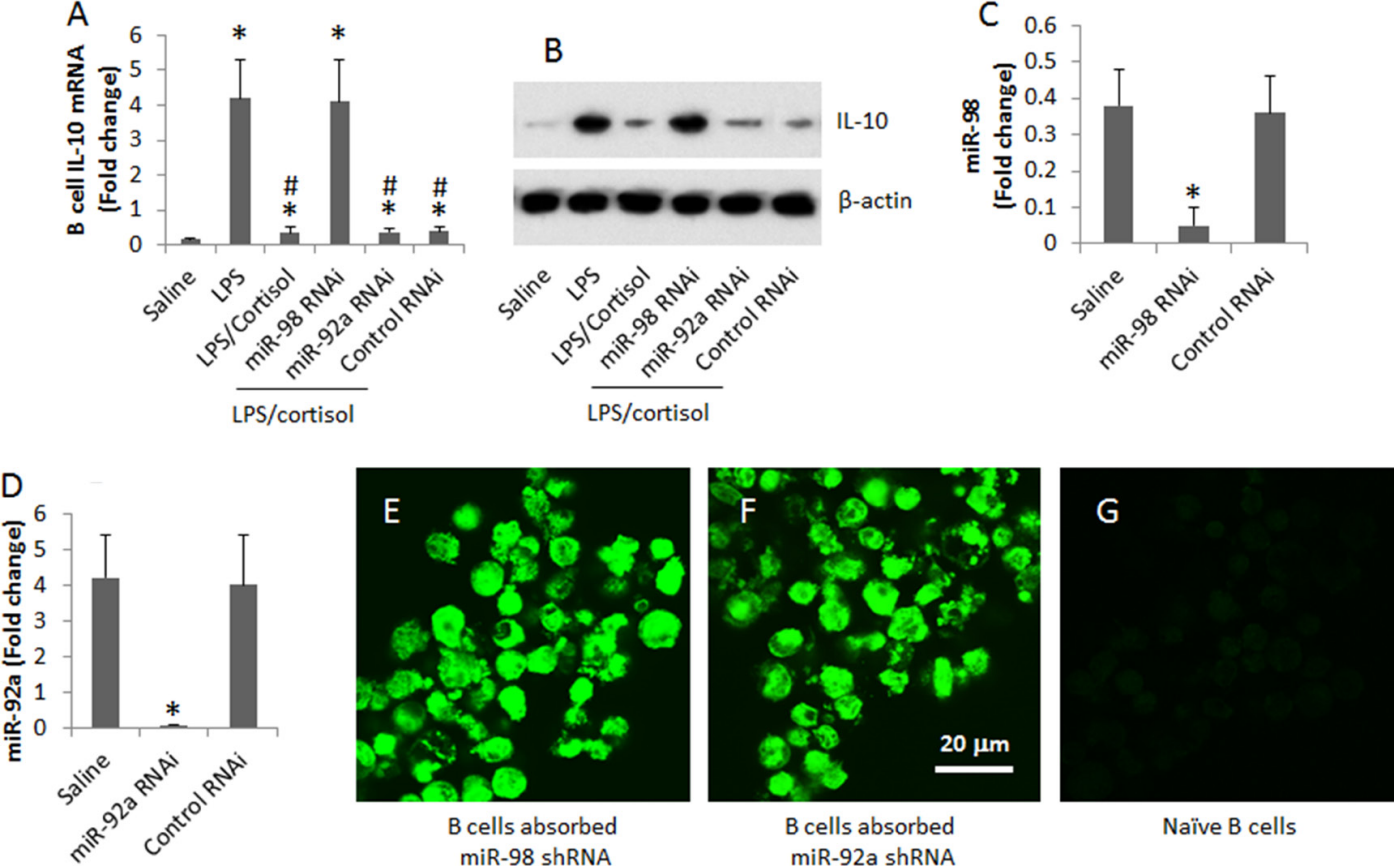
Assessment of the effects of miR-98 on suppression of IL-10 expression in B cells (**A**, **B**) the levels of IL-10 mRNA (A) and protein (B) in B cells after treatment in the culture as denoted on the X axis. (**C**) the bars show the results of miR-98 RNAi of B cells. (**D**) the bars show the results of miR-92a RNAi of B cells. Data of bars are presented as mean ± SD. *p < 0.01, compared with the saline group. (**E**, **F**) representative images show that B cells were transduced with miR-98 shRNA (E) or miR-92a shRNA (F) (the shRNA carried a GFP gene; the green color indicates the GFP protein). (**G**) an image of naive B cells. The data were summarized from 3 independent experiments. Original magnification of E–G: '630.

By employing an established cell culture model [17], we were able to up regulate IL-10 expression in B cells. Peripheral B cells were isolated from blood samples collected from healthy subjects. The B cells were stimulated with LPS (to up regulate the expression of IL-10) with or without the presence of cortisol in the culture for 3 days. As analyzed by RT-PCR, cortisol suppressed the expression of IL-10 in B cells in a cortisol dose-dependent manner. To corroborate the results, we prepared the miR-deficient B cells by transducing B cells with miR-98 shRNA-laden lentivirus or control lentivirus. The transduction resulted about 10 folds down of the miR-98 expression in the B cells. The miR-98-deficient and wild B cells were exposed to LPS or/and cortisol in the culture for 48 h. The B cells were analyzed by RT-qPCR. The results showed that the knockdown of miR-98 reduced about 10 folds of the effects of cortisol on suppression of IL-10 in B cells. To test if non-specific miRs could interfere with the expression of IL-10 in B cells, we knocked down the expression of miR-92a, in line with other's reports [18], which did not affect the IL-10 expression in B cells (Figure 4).

### Therapies of anti-miR-98, or cortisol inhibitor, or adoptive transfer with B10 cells enhance the allograft heart survival in mice

**Figure 5:**
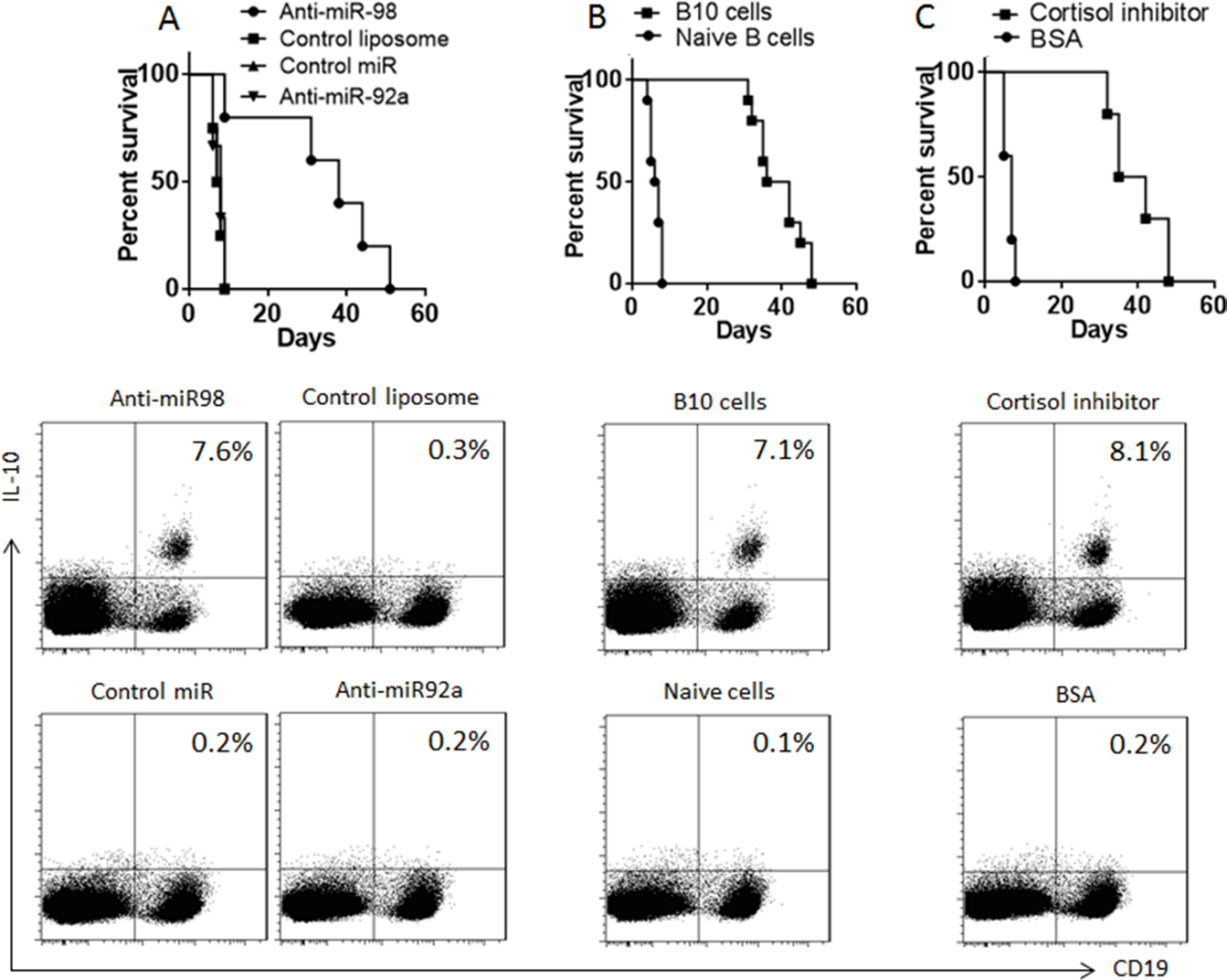
Survival allograft hearts (**A**–**C**) the survival curves show the survival rates of mice received allograft heart transplantation. The flow cytometry plots show the frequency of B10 cells in the mouse spleen after heart transplantation. The treatments are denoted above each panel. Anti-miR-98 (anti-miR-92a, or control miR): Mice were also anti-miR-98 liposomes (or anti-miR-92a liposomes, or control liposomes). B10 cells (Naïve cells): Mice received B10 cells (or naïve B cells) at 10^6^ cells/mouse one day before surgery. Cortisol inhibitor (B), or B10 cells (C), or cortisol inhibitor or control materials. Each group consists of 10 mice.

Data reported above suggest that anti-miR-98, or cortisol inhibitor, or adoptive transfer with B10 cells might enhance the allograft heart survival. To test this, we performed heart transplantation in mice. The mice were also received intraperitoneal injection with anti-miR-98 liposomes (or control liposomes; or anti-miR-92a liposomes) (Figure 5A), or adoptive transfer with B10 cells (or naïve B cells) (Figure 5B), or 33851 (An 11β-HSD1 inhibitor used as a cortisol inhibitor, or BSA) (Figure 5C). The results showed that mice received saline, or control reagent/cells, lived less than 10 days, while those treated with anti-miR-98 liposomes, or 33851, or B10 cells, lived more than 30 days (Figure 5).

## DISCUSSION

In this study, we observed that the expression of IL-10 was compromised in peripheral B cells in patients after heart transplantation, indicating that the immune tolerance system may be affected by the heart transplantation. The surgery of heart transplantation can result in the effects of psychological stress [19]. By activating the pituitary-peripheral system axis, the “stress hormones” are released to the peripheral system to modulate physiological functions. These hormones include cortisol, noradrenaline or adrenaline, etc. The present data show these hormones in the peripheral system of patients after surgery. Others also observed similar reactions in the pituitary-peripheral axis; such as Yang et al found that psychological stress unusually increased the intestinal epithelial barrier permeability to protein antigens and to initiate the intestinal immune inflammation [20]. Emotional reaction to surgical operation was also reported. Yang et al observed a group of cancer patients had high levels of peripheral corticotrophin releasing factor pre-surgical operation [21].

Our data show that serum cortisol levels are negatively correlated with the frequency of peripheral B10 cells. Following this hint, we found that exposure to cortisol in the culture suppressed the expression of IL-10 in B cells. The data implicate that the heart transplantation surgery results in enhancement of serum cortisol levels and the cortisol down regulates the expression of IL-10 in B cells. Others also found that stress response suppressed IL-10 [22]. IL-10 is an important immune regulatory molecule that inhibits the immune response of other immune effector cells [23] and contributes to immune tolerance [24]. The results suggest that it is necessary to elucidate the mechanism by which cortisol suppresses the expression of IL-10 in B cells.

Published data indicate that miR-98 can bind to the 3′-untranslated regions of IL-10 and thereby result in IL-10 gene repression by inhibiting translation and destabilizing transcripts [15, 25]. Our data also show that higher levels of miR-98 were detected in the peripheral B cells of patients after heart transplantation, which is positively correlated with the serum cortisol levels and negatively correlated with the IL-10 mRNA levels in B cells. The fact suggests that miR-98 may mediate the effects of cortisol on suppression of IL-10 in B cells. The inference is supported by subsequent data. Exposure to cortisol in the culture markedly increased the expression of miR-98 in B cells and the cortisol-suppressed IL-10 expression in B cells could be blocked by knocking down the miR-98 gene as we observed in the present study.

To establish immune tolerance can promote the allograft survival. Zhang et al indicate that donor CD47 plays an important role in the control of T-cell alloresponses and tolerance induction following hepatocyte transplantation via suppression of effector T cell expansion [26]. Li et al indicate that transfecting dendritic cells with indoleamine 2,3-dioxygenase (IDO) significantly improves the allograft survival in cardio transplantation via regulating the tryptophan catabolism [27]. In general, it is considered that B cells are detrimental to organ transplantation based on their capacity to produce the donor specific antibodies. However, the transitional and tolerant B cells can be beneficial to organ transplantation [28]. The present study has made a further step in this area by employing three approaches, including anti-miR-98, cortisol inhibitor and adoptive transfer with B10 cells, in mouse heart transplantation. The results showed that all the three approaches resulted in significant improvement in the allograft heart survival.

In this study, the patients received immunosuppressive therapy after heart transplantation. The immunosuppressive agents affect effector T cell function [29]; whether they also suppress B10 cells is to be further investigated.

In summary, the present data show that after heart transplantation, the peripheral cortisol levels were increased, which suppressed the expression of IL-10 in B cells and may contribute to the reduction of peripheral B10 cells. Inhibition of miR-98, or administration of cortisol inhibitor, or adoptive transfer with B10 cells, significantly improved the allograft heart survival, suggesting that these approaches have the potential to be used in clinical heart transplantation.

## MATERIALS AND METHODS

### Patients and ethic statement

Patients (10 male and 10 female, age: 25–66 years old) with advanced heart failure and scheduled to receive allograft heart transplantation were recruited into this study. Their demographic data are presented in Table 1. The determination of heart transplantation was made by both the patients and our surgeons following the routine procedures in our hospital that were also published by other investigators [30]. Treatment with immunosuppressors (cyclosporine + azathioprine) was carried out after the transplantation. The study procedures were approved by the Human Ethic Committee at Fuwai Hospital. An informed written consent was obtained from each patient.

**Table 1:** Baseline characteristics of the patients

Characteristics	Values
Number	20
Demography	
Age (year)	54.2 ± 15.8
Male (no. %)	10 (50)
Body-mass index	21.1 ± 3.8
Clinical characteristics	
Diagnosis to transplantation (months)	30.8 ± 33.1
SBP (mmHg)	96.5 ± 11.8
Heart rate	79.1 ± 14.9
Medication history	
Antiplatelet (no.%)	4 (20)
Digoxin (no. %)	16 (80)
ACEI/ARB (no.%)	10 (50)
Diuretic (no.%)	20 (100)
β-blockers (no.%)	16 (80)
Antiarrhythmic (no.%)	10 (50)
Cardiac function	
I (no.%)	0
II (no.%)	5 (25)
III (no.%)	0
IV (no.%)	15 (75)
Hypertension	0
Diabetes	0
Hyperlipidemia	4 (20)
ECG	
AF	5 (25)
LBBB	0
RBBB	4 (20)
PVT	4 (20)
UCG	
LAD (mm)	46.9 ± 5.8
LVEDD (mm)	67.9 ± 5.9
EF (%)	31.3 ± 5.6
MR (%)	20 (100)

ECG: electrocardiogram; AF: atrial fibrillation; LBBB: left bundle branch block; RBBB: right bundle branch block; PVT: paroxysmal ventricular tachycardia; UCG: ultrasonic cardiogram; LAD: left atrial diameter; LVEDD: Left ventricular end diastolic diameter; EF: Ejection fraction; MR: Mitral regurgitation (moderately to severe).

### Collection of peripheral blood samples

Peripheral blood samples (30 ml per subject) were collected from each patient before surgery, 3 days and 2 weeks after surgery, and 20 healthy subjects (10 male and 10 female, age: 20–60 years old), via ulnar vein puncture.

### Isolation of peripheral B cells

The peripheral blood mononuclear cells (PBMC) were isolated from the blood samples by gradient density centrifugation. CD19^+^ B cells were purified from the LPMCs by magnetic cell sorting (MACS) with a reagent kit (Miltenyi Biotech) following the manufacturer's instructions. The purity of the isolated B cells was greater than 98% as checked by flow cytometry.

### Cell culture

The isolated B cells were cultured in RPMI1640 medium supplemented with 10 fetal bovine serum, 0.1 mg/ml streptomycin, 100 U/ml penicillin, 2 mM L-glutamine and anti-CD40 mAb (20 ng/ml; to avoid B cell apoptosis). The medium was changed in 2–3 days.

### Assessment of peripheral B10 cells by flow cytometry

PBMCs were stained with FITC-labeled anti-CD19 antibody (or isotype IgG) (BD Bioscience) for 30 min at 4°C, washed with phosphate-buffered saline (PBS), fixed with 1% paraformaldehyde for 1 h, incubated with 0.5% saponin (Sigma Aldrich), stained with APC-labeled anti-IL-10 antibody (or isotype IgG) for 30 min at 4°C, washed with PBS, and analyzed with a flow cytometer (FACSCanto II; BD Bioscience). The data were analyzed with Flowjo (TreeStar, Ashland OR). Data from isotype IgG-stained cells were used as a gating reference.

### Urine collection

The 24-h urine/day was collected from each patient before surgery and 1–8 day after surgery.

### Measurement of cortisol, noradrenaline and adrenaline

The levels of cortisol, noradrenaline and adrenaline in the urine, and the cortisol levels in the sera, were determined by enzyme-linked immunosorbent assay (ELISA) with commercial reagent kits (Biomart, Beijing, China) following the manufacturer's instructions.

### Assessment of miR-98 and IL-10 mRNA in B cells by real time RT-PCR (RT-qPCR)

The total RNAs were extracted from purified B cells with the TRIzol reagents (Invitrogen). The cDNA was synthesized from the RNA with a reverse transcription kit (Invitrogen). The samples were amplified in a real time PCR device (Mini-Opticon, Bio-Rad, Hercules, CA) with the SYBR Green Master Mix (Invitrogen). The primers of miR-98 were provided by Enke Biotech (Shenzhen, China). Reference gene RNA U6B (Invitrogen) was analyzed as an internal control. The sequences of IL-10 primers for PCR are gttctttggggagccaacag and gctccctggtttctcttcct. The results were calculated by the 2^−ΔΔCt^ method and presented as fold change against controls.

### Assessment of effects of cortisol on expression of miR-98 in B cells

B cells were isolated from blood samples collected from healthy persons and cultured (10^6^ cells/ml) in the presence of cortisol (Sigma Aldrich) at gradient concentrations for 3 days. The expression of miR-98 in B cells was assessed by RT-qPCR.

### Preparation of miR-98- or miR-92a-deficient B cells

B cells were transduced with miR-98 shRNA or miR-92a shRNA carried by lentivector or control lentivector (Enke Biotech, Shenzhen, China) following the manufacturer's instructions. Briefly, cells were transduced with miR-98-shRNA or miR-92a-shRNA or control shRNA lentivector-containing media (2.0 × 10^6^ viral particles/ml). The media were changed 24 h later; the cells were then incubated for another 16 hours. GFP expression was imaged using a confocal microscope (LSM710, Carl Zeiss) at ×63 magnification with a fluorescein isothiocyanate filter. The effects of gene knockdown were assessed by RT-qPCR.

### Assessment of effects of miR-98 on expression of IL-10 in B cells

MiR-98-sufficient or miR-98-deficient B cells were incubated in the presence of LPS (lipopolysaccharide; Sigma Aldrich; 1 μg/ml) with or without the presence of cortisol for 3 days. The expression of IL-10 in the B cells was assessed by RT-qPCR.

### Promoting allograft heart survival in mice with anti-miR-98, or cortisol inhibitor, or adoptive transfer with B10 cells

### Preparation of anti-miR-98 liposomes

Following published procedures [31], anti-miR-98 or anti-miR-92a oligonucleotides (0.086 μmol; Enke Biotech, Shenzhen, China) were mixed with a lipid mixture (Sigma Aldrich) in 200 μL of 100% ethanol and 300 μl of 20 mM citrate buffer (pH 4) at 60°C. The samples were extruded through a polycarbonate membrane (100-nm-diameter) using a LiposoFast basic extruder (Avestin, Toronto, Canada). A Sepharose CL-4B column was prepared and equilibrated with HBS pH 7.4. The samples were passed through the column to remove ethanol and nonencapsulated anti-miR-98. The total lipid concentration was assessed by cholesterol quantification using the Liebermann–Burchard test [31].

### Generation of B10 cells

**Figure 6:**
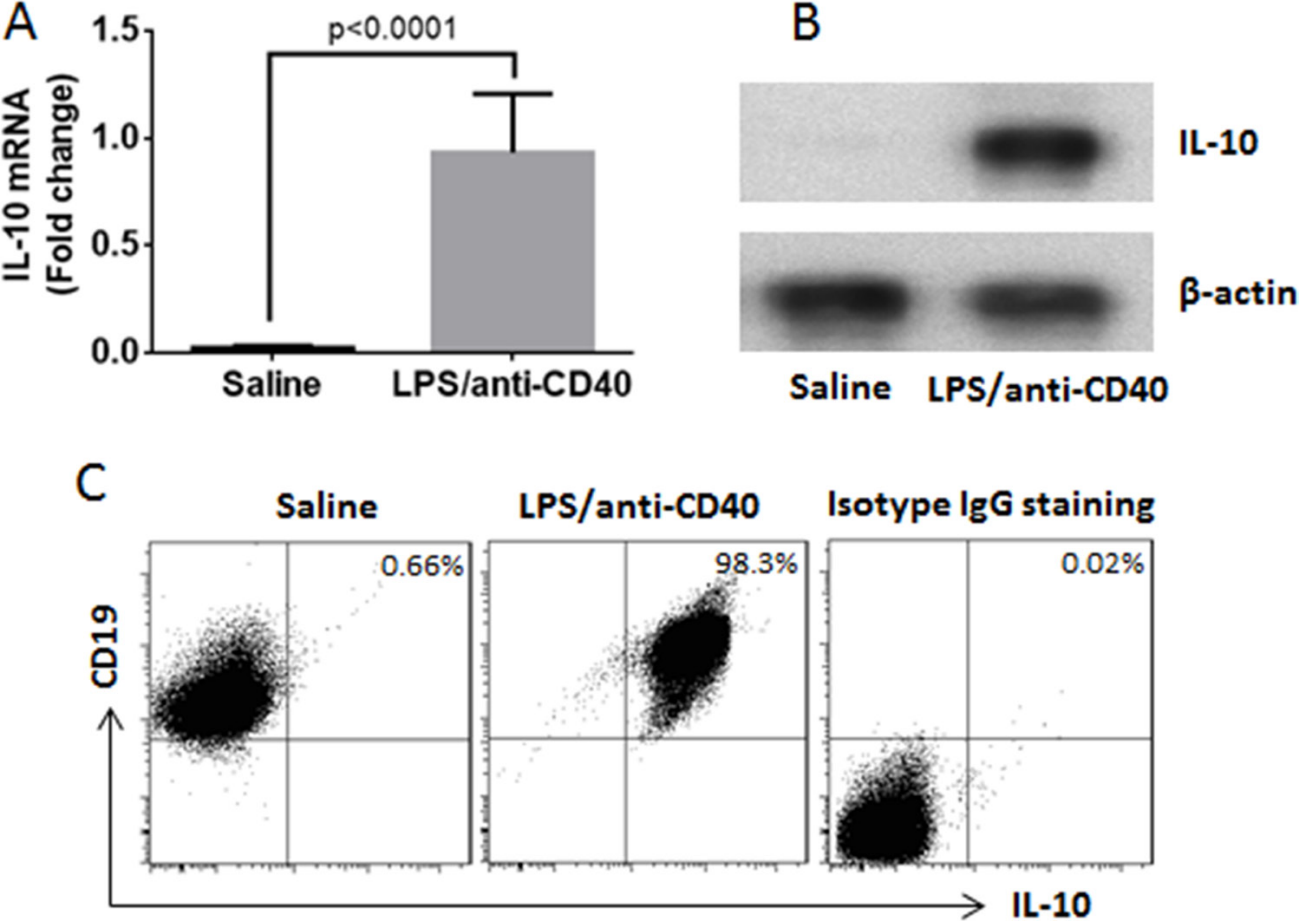
Induction of B10 cells Naive B cells were isolated from the mouse spleen and cultured for 3 days in the presence of saline, or LPS (1 μg/ml) and anti-CD40 antibody (20 ng/ml). (**A**) the IL-10 mRNA in the B cells (by RT-qPCR). (**B**) the IL-10 protein in B cells (by Western blotting). (**C**) the frequency of B10 cells (by flow cytometry). The data represent 3 independent experiments.

CD19^+^ B cells were isolated from the naïve mouse spleen by MACS (the purity was greater than 99% as checked by flow cytometry). The B cells were cultured at 10^6^ cells/ml in the presence of LPS (10 μg/ml) and anti-CD40 mAb (20 ng/ml) for 3 days. As checked by flow cytometry, the IL-10^+^ B cells were more than 98% (Figure 6).

### Allograft heart transplantation in mice

Male BALB/c mice and male C57/B6 mice were purchased from the Beijing Experimental Animal Center. The mice were maintained in a pathogen-free environment and allowed to access food and water freely. The using mouse in the present study was approved by the Animal Ethic Committee at Fuwai Hospital (approve number: FW20150012).

The hearts from BALB/c mice were transplanted into C57/B6 mice, which were carried out following our established procedures [32]. Briefly, under general anesthesia, the donor heart was removed under a sterile environment. The donor aorta was anastomized to the recipient aorta and the donor pulmonary artery was anastomized to the recipient inferior vena caca using 10–0 nylon suture.

### Promoting allograft heart survival

One day prior to surgery, mice were treated with one of the following procedures: (A) Intraperitoneal injection with anti-miR-98 liposome (0.1 mg/mouse; or control liposomes; or anti-miR-92a liposomes. The liposomes were prepared by the Enke Biotech, Shenzhen, China). (B) Adoptive transfer with B10 cells or naïve B cells at 10^6^ cells/mouse via tail vein injection one day before surgery. (C) Intraperitoneal injection with 11β-HSD1 inhibitor, 385581 (5 mg/kg; used as an inhibitor of cortisol. Merck, Darmstadt, Germany. Or BSA (0.1 mg/muse) using as a control reagent). The survival dates were recorded for each mouse.

### Statistical analysis

The data are normally distributed as analyzed by the Norm. DIST in Microsoft Excel and expressed as mean values ± standard deviation (SD). The Student's *t*-test was used to determine the level of significance of differences between the two groups or ANOVA along with Bonferroni correction if more than two groups. Correlation assay was performed with Microsoft Excel. Statistical significance was defined as *P* < 0.05.
